# The efficacy of botanical drugs in orchestrating epigenetic modifications for ameliorating metabolic disorders

**DOI:** 10.3389/fphar.2024.1366551

**Published:** 2024-04-05

**Authors:** Eko Fuji Ariyanto

**Affiliations:** Department of Biomedical Sciences, Faculty of Medicine, Universitas Padjadjaran, Bandung, Indonesia

**Keywords:** botanical drugs, epigenetics, DNA methylation, histone modifications, non-coding RNA, metabolic disorder

## Introduction

Metabolic diseases remain the most worrying global health problem as their prevalence is increasing globally every year (Choi et al., 2008). Moreover, most metabolic diseases and their complications including coronary artery disease, diabetes mellitus, hypertension, and obesity are chronic and associated with a very high financial burden. Consequently, various efforts have been made to develop safe drugs with mild side effects for long-term consumption.

Epigenetics are changes in gene expression without changes in DNA sequences and plays a role in cellular processes in various diseases (Ling and Ronn, 2019; Guo et al., 2021). Epigenetic changes can be caused by at least three mechanisms including DNA methylation, post-translational histone modifications, and regulation of gene expression by non-coding RNA (ncRNA) which can be microRNA (miRNA), long non-coding RNA (lncRNA), etc (Xiao et al., 2019). Increased DNA methylation at gene promoters causes decreased gene expression, increased ncRNA activity reduces gene expression through degradation of messenger RNA (mRNA), while the effect of histone modifications on gene expression is more variable depending on the type and location of substrate attachment to the histone tail (Xiao et al., 2019). Epigenetics plays an important role in the development of many metabolic diseases (Tikoo et al., 2008; Stančáková and Laakso, 2016; Ling and Ronn, 2019; Rizzacasa et al., 2019; Samblas et al., 2019; Wu et al., 2023) and since they are reversible, restoring epigenetic status to normal provides opportunities for developing pharmacotherapies for metabolic diseases (Hamm and Costa, 2015).

Currently, botanical drug is an interesting topic of discussion for the treatment of metabolic diseases because of its low toxicity and promising therapeutic effects (Ariyanto et al., 2023a; Ariyanto et al., 2023b; Wu et al., 2023). Botanical drugs are composed of many compounds, some of which provide therapeutic effects (Ariyanto et al., 2023a; Ariyanto et al., 2023b; Wu et al., 2023) and are usually consumed in the form of decoction, pills, powder, etc. extracted from the leaves, fruit, roots, or stems of plants (Wu et al., 2023).

Several studies have determined the effect of botanical drugs on metabolic diseases but its effectiveness in modifying the epigenetic status of molecular pathways involved in the pathogenesis and pathophysiology of metabolic diseases has not yet been revealed. This article aims to comprehensively analyze the efficacy of botanical drugs in treating metabolic diseases through epigenetic changes to provide insight into research and development strategies for botanical drugs as a pharmacotherapy for metabolic diseases.

Previous studies have shown that botanical drugs can produce epigenetic changes through several mechanisms including modulating DNA methylation, posttranslational histone modifications, as well as ncRNA-mediated gene regulation by modulating the expression or activity of DNA methyltransferase (DNMT) and histone deacetylase (HDAC) (Wu et al., 2023). DNMT catalyzes DNA methylation while HDAC catalyzes the release of acetyl groups from histones to inhibit gene expression (Verdin et al., 2003; Mirza et al., 2013).

## Botanical drugs that modulate DNA methylation

Several studies have reported the effect of botanical drugs in changing DNA methylation levels to improve metabolic disease conditions. Zhou showed that geniposide present in *Hedyotis diffusa*, *Radix scrophulariae*, *Eucommia ulmoides*, and *Paederia scandens* has antiatherosclerotic effects through regulating DNA methylation (Zhou, 2019). Ma *et al* showed that resveratrol has important benefits in preventing cardiovascular disease because it can inhibit homocysteine-induced PTEN hypermethylation to inhibit smooth muscle cell proliferation, one of the stages of atherogenesis (Ma et al., 2018). Another study indicated the role of curcumin in increasing the methylation of the RNA18S5 gene through activation of DNMT2 which produces atheroprotective effects (Lewinska et al., 2015). An *in vivo* study reported the role of naoluoxintong (NLXT) in a rat model of ischemic stroke with middle cerebral artery occlusion (MCAO) (Hong et al., 2021). NLXT regulated *NogoA*, *NgR1*, *NgR2*, *RhoA,* and *Rock2* gene expression through downregulation of DNA methylation (Hong et al., 2021).

## Botanical drugs that modulate posttranslational histone modifications

Resveratrol, a metabolite contained in melinjo seeds, improved outcomes in type 2 diabetes mellitus patients through epigenetic modification (Bo et al., 2018). Administering 40 mg and 500 mg for 6 months to type 2 diabetes mellitus patients increased Sirtuin-1 (Sirt1) expression which was associated with a decrease in H3K56 acetylation and body fat (Bo et al., 2018). Naringenin and hesperetin, Quzhou Fructus aurantia-derived metabolites, inhibited AMPK-mediated p300 induction to decrease histone acetylation, thereby decreasing *Txnip* expression in pancreatic β cells in diabetic mice and the INS-1 pancreatic β cell line (Wang et al., 2021).

Several *in vivo* studies reported the potential of esculetin, a derivative of coumarin, in improving diabetes mellitus and its complications through epigenetic modification (Kadakol et al., 2015; Kadakol et al., 2017). Esculetin administered at doses of 50 and 100 mg/kg/day for 2 weeks reduced dimethylation at lysine 4 of histone 3 (H3K4me2) and H3K36me2, as well as acetylation at lysine 27 of histone 3 (H3K27ac) in the hearts of type 2 diabetes mice (Kadakol et al., 2015). The administration of 50 mg/kg/day esculetin for 6 weeks also improved cardiomyopathy caused by type 2 diabetes mellitus by reducing levels of H3K9ac, H2AK119ub, and H2BK120ub (Kadakol et al., 2017). Therefore, esculetin has the potential to be developed for the treatment of diabetes mellitus and its complications through epigenetic modifications, especially in histones.

*In vivo* studies in mouse models and *in vitro* studies reported that icariin pretreatment (4 μM) prevented ischemia/reperfusion (I/R)-induced injury by increasing the activity of sirtuin-1, a histone deacetylase, which then reduced FOXO1 (Wu et al., 2018). This mechanism improved the quality of heart contractions, limited the size of cardiac infarction, and leakage of creatine kinase-MB from damaged myocardium, as well as reduced oxidative stress in heart cell mitochondria (Wu et al., 2018). Moreover, administration of sirtuin-1 inhibitors and *Sirt1* siRNA reduced the visible cardioprotective effects (Wu et al., 2018).

Suxiao Jiuxin pill (SJP) is an botanical drug that contains tetramethylpyrazine and borneol and has often been used as a therapy for coronary artery disease in China (Ruan et al., 2018). In the context of cell-cell communication in the heart, exosomes play a pivotal role in cardiac mesenchymal stem cell and cardiomyocyte communication, some of which can occur through the modulation of epigenetic changes (Ruan et al., 2018). Ruan *et al* showed that SJP treatment can cause changes in C-MSC-derived exosomes to increase H3K27me3 and decrease *Utx* expression, as well as increase *Pcna* expression, a marker of cardiomyocyte proliferation in HL-1 cells, a mouse cardiomyocyte line (Ruan et al., 2018).

Anacardic acid was reported to have an inhibitory effect on histone acetylation in a cardiac hypertrophy mice model. Administration of 3.75 mg/kg anacardic acid improved cardiac hypertrophy through modulating histone acetylation (Li et al., 2019) by inhibiting the expression of p300 and p300/CBP-associated factor (PCAF), thereby reducing H3K9ac levels and normalizing the transcriptional activity of Mef2 (Li et al., 2019). Anacardic acid also inhibits the activity of histone acetylases in mouse cardiac hypertrophy, causing changes in the expression of several important genes in the heart and reducing cardiac hypertrophy (Peng et al., 2017).

*In vitro* studies reported the effectiveness of kaempferol and piperine in inhibiting adipocyte differentiation and increasing lipolytic gene expression, respectively, through epigenetic modification (Park et al., 2019; Park et al., 2022). Administration of 100 μM kaempferol inhibited the expression of several *Pparg* target adipogenic genes including *Adipoq*, *Fabp4*, and *Lpl* by reducing H3K27me3 deposition in the gene promoter region during 3T3-L1 adipocyte differentiation (Park et al., 2022). Administration of 50 μM piperine for 8 days to the 3T3-L1 cell line decreased H3K27me3 enrichment in *Pparg*, decreased H3K9ac, and increased *Ezh2*, increasing the expression of *Ezh2*-associated lipolytic genes (Park et al., 2019).

Qian Yang Yu Yin (QYYY) granules improve renal injury conditions through epigenetic regulation in HEK293T cells whose proliferation was induced by Ang II as a renal damage model (Zhang et al., 2020). QYYY inhibits the proliferation of HEK293T cells, acetyl-cortactin, and DNA methylation, as well as increasing *Sirt1* and H3K4me3 (Zhang et al., 2020).

## Botanical drugs that modulate ncRNA

Several *in vivo* studies demonstrated the role of botanical drugs in improving the pathological conditions of metabolic diseases through miRNA regulation. Supplementation with plant-derived polyphenols reduces weight gain, liver steatosis, insulin resistance, and histopathological damage in diet-induced fatty liver disease in hyperlipidemic mice through regulation of the miRNA paralogs miR-103/107 and miR-122 which then modulated glucose and lipid metabolism (Joven et al., 2012). Supplementation with 0.05% lycopene for 8 weeks inhibited liver steatosis in high-fat-fed mice through miRNA-21 induction, which then caused *Fabp7* degradation and decreased fatty acid-binding protein 7 (FABP7) expression (Ahn et al., 2012). Another *in vivo* study unraveled that the administration of 40 mg/kg Xuesaitong increased myocardial blood vessel formation in a myocardial infarction mouse model through inhibiting miR-3158-3p targeting *Nur77* (Liao et al., 2023).

Yang *et al* showed the effect of administering 25 μmol/L dihydromyricetin in increasing endothelial nitric oxide (NO) synthesis and inhibiting atherosclerosis through inhibiting miR-21 in the apolipoprotein E-deficient mice model (Yang et al., 2020). Inhibition of miR‐21 further increased the expression of the gene encoding dimethylarginine dimethylaminohydrolase‐1, which in turn decreased asymmetric dimethylarginine and increased endothelial NO synthase to increase NO production (Yang et al., 2020). *In vivo* research on a rat myocardial infarction model showed that Wenxin granules improved myocardial infarction by regulating miR-1 and protein kinase C (PKC)-mediated signal transduction which protected gap junctions and increased the ventricular fibrillation threshold (Wu et al., 2017).

A study using human coronary artery endothelial cell-derived exosomes found that chrysin treatment reduced endothelial cell-derived miR-92a-containing exosomes which then caused an increase in *Klf2* expression for an atheroprotective effect (Lin et al., 2021). Yunpi Heluo decoction improved insulin resistance in Zucker diabetic fatty rats by reducing miR-29a-3p expression causing increased *Irs1* expression, a target of miR-29a-3p (Mao et al., 2019), thereby increasing the protein expression of IRS1, protein kinase B, and pyruvate dehydrogenase lipoamide kinase isozyme 1 (PDK1) (Mao et al., 2019). Another study found that quercetin, perillyl alcohol, and berberine improved pathological conditions in pulmonary arterial hypertension by regulating miR-204 and miR-27a, as well as factors that play a role in inflammation, fibrosis, and apoptosis (Rajabi et al., 2021). Moreover, Cao *et al* suggested the role of *Astragalus polysaccharide* in accelerating the differentiation of C3H10T 1/2 cells into brown adipocytes through the regulation of miR-1258-5p and *Cux1* (Cao et al., 2021).

## Discussion

The burgeoning field of botanical drugs has garnered significant attention in recent years due to its perceived potential and effectiveness in the treatment of metabolic diseases. Several investigations and empirical data discussed earlier provide detailed insights into the potential and effectiveness of botanical drugs in managing metabolic diseases. Several studies have indicated that even at low doses, botanical drugs can yield significant effects, suggesting considerable efficacy.

Despite the promising outcomes observed in the previous studies, the precise molecular mechanisms underlying the therapeutic effects of botanical drugs remain incompletely understood. A key area of inquiry pertains to the epigenetic alterations and gene regulation induced by secondary metabolites. Delving deeper into these mechanisms necessitates elucidating, for instance, the specific binding proteins or transcription factors involved in mediating posttranslational histone modifications, modulating gene expression, and, subsequently, producing biological effects. This intricate interplay between secondary metabolites and molecular pathways warrants further exploration through more advanced molecular and biochemical analyses.

Moreover, alongside unraveling the molecular intricacies, it is crucial to conduct rigorous investigations into the safety profile of botanical drugs. While botanical drugs offer potential therapeutic benefits, ensuring their safety is paramount. Comprehensive toxicity studies and pharmacological evaluations are essential to ascertain any potential adverse effects associated with prolonged usage or interactions with other medications and compounds. Such thorough assessments are fundamental for mitigating risks and promoting the responsible use of botanical drugs in clinical settings.

Furthermore, the exploration of potential side effects and drug interactions extends beyond individual metabolites to encompass their synergistic effects and interactions with conventional pharmaceuticals. Understanding how botanical drugs interact with other molecules, including prescription drugs, is essential for preventing adverse reactions and optimizing therapeutic outcomes. Integrating pharmacokinetic and pharmacodynamic studies can provide valuable insights into the bioavailability, metabolism, and potential drug interactions of herbal formulations.

While empirical evidence highlights the therapeutic potential of botanical drugs in managing metabolic diseases, further research is imperative to elucidate the underlying molecular mechanisms and ensure their safety and efficacy. By leveraging advanced scientific methodologies and conducting comprehensive evaluations, we can unlock the optimal therapeutic potential of botanical drugs while safeguarding patient health and wellbeing.

## Conclusion

Botanical drugs in relatively small doses produce beneficial effects in various pathological conditions involved in metabolic diseases by changing the level of DNA methylation or post-translational histone modifications, or modulating ncRNAs. However, further studies elaborating more specific molecular mechanisms, safety, adverse effects and potential interactions with other molecules are required to accelerate the development of novel drugs.
